# Textured Sr_2_Sc_0.1_Nb_0.1_Co_1.5_Fe_0.3_O_6−2δ_ Thin Film Cathodes for IT-SOFCs

**DOI:** 10.3390/ma12050777

**Published:** 2019-03-07

**Authors:** Zhaoxin Zhu, Chuan Zhou, Wei Zhou, Nan Yang

**Affiliations:** 1 School of Physical Science and Technology, ShanghaiTech University, 393 Middle Huaxia Road, Pudong, Shanghai 201210, China; zhuzhx@shanghaitech.edu.cn; 2 Shanghai Institute of Ceramics, Chinese Academy of Sciences, Shanghai 200050, China; 3 University of Chinese Academy of Sciences, Beijing 100049, China; 4 Jiangsu National Synergetic Innovation Center for Advanced Materials (SICAM), State Key Laboratory of Materials-Oriented Chemical Engineering, College of Chemical Engineering, Nanjing Tech University, No.5 Xin Mofan Road, Nanjing 210009, China; zhouchuan987654@163.com

**Keywords:** SOFCs, mixed ionic electronic conductor, ORR, thin films, surface chemistry

## Abstract

Reducing the operating temperature of solid oxide fuel cells (SOFCs) to intermediate (650–850 °C) or even lower levels (400–650 °C) is an important practical requirement. However, the main obstacle to lowering the operating temperature is the poor oxygen reduction reaction (ORR) activity on the cathode side and, therefore, it is essential to explore cathode materials with good ORR activity in these temperature ranges. In this work, we investigated the possibility of using Sr_2_Sc_0.1_Nb_0.1_Co_1.5_Fe_0.3_O_6−2δ_ (SSNCF) as a suitable intermediate temperature cathode material. SSNCF thin films with different orientations were prepared using the pulsed laser deposition technique, and the relationship of the surface chemical states and ORR activity was discussed in terms of crystallographic orientation. The results showed that the SSNCF/YSZ grown along the [110] direction exhibited superior ORR activity compared to the SSNCF/SDC/YSZ thin film electrode grown along the [100] direction. This was explained by the variation in the Sr-surface enrichment and cobalt ion oxidation state using X-ray photoemission spectroscopy.

## 1. Introduction

Solid oxide fuel cells (SOFCs) are a type of device that converts the chemical energy from fuel directly into electrical energy without the need for combustion. SOFCs have been continuously investigated for their simple structure, high energy conversion efficiency, fuel flexibility, and zero exhaust gas emission (i.e., SO_2_, NO). However, the high operating temperature (>850 °C) results in high maintenance costs and material compatibility issues. Over the past decade, significant progress has been made to bring the operating temperature down to the intermediate (650–850 °C) and even lower ranges (400–650 °C) [1]. When the temperature is below 650 °C, the polarization resistance (Rp) of the oxygen reduction reaction (ORR) (O_2_ + 4e^−^→2O^2−^) on the cathode increases significantly, due to the sluggish reaction kinetics that occur at lower temperatures [2,3]. Continuous improvements to cathode materials have been made, specifically, mixed ionic and electronic conducting (MIEC) oxides with a perovskite structure, such as La_1−x_Sr_x_CoO_3−δ_ and Ba_1−x_Sr_x_Co_1−y_Fe_y_O_3−δ_, have been widely proposed as cathode materials for SOFCs [4]. MIEC oxides not only provide electrons for the oxygen reduction reaction, but they also facilitate the transfer of oxygen ions to the electrolyte. As a result, the ORR is no longer limited to the three-phase-boundary of the cathode, electrolyte, and gas molecules, as the active sites can now be enlarged to the whole cathode surface [5].

SrCoO_3−δ_-based solid solutions with high oxygen ion conductivity and good ORR activity related to the Co^3+^/Co^4+^ redox couple have also attracted wide attention for improving the cathode performance at low temperatures [6,7]. Chemical doping strategies were adopted to further tailor the SrCoO_3−δ_ based cathode performances. For example, by substituting Co with donor dopants, such as Sc and Nb ions, an enhancement in the electronic conductivity can be achieved. In addition, doped SrCoO_3−δ_ based cathodes possess a better phase stability under thermal cycling and long-term working conditions compared to the parental phase [8,9]. Furthermore, replacing Co ions with a small amount of Fe ions can improve the overall cell performance. In addition to the enhanced ORR activity, Fe-doped SrCoO_3−δ_-based cathodes demonstrate a lower thermal expansion coefficient and better CO_2_ tolerance. According to related work, these properties could be also related to the oxygen non-stoichiometric effect [10,11]. Recently, Chuan Zhou et al. prepared a novel kind of doped SrCoO_3−δ_ cathode, i.e., Sr_2_Sc_0.1_Nb_0.1_Co_1.5_Fe_0.3_O_6−δ_ (SSNCF). By simultaneously doping Sc, Nb, and Fe, both the electronic and ionic conductivity could be improved, making SSNCF a potential candidate for intermediate temperature application [12]. Although Sc-, Nb-, and Fe-doped SrCoO_3−δ_ cathode materials were intensively investigated, the most reported works were performed using samples comprising of ceramics pellets and thick polycrystalline films [13,14]. However, owing to the complicated effects caused by grain boundaries, defect densities, and crystal grain sizes, their intrinsic activities were not completely explored. Furthermore, some properties related to the surface states, such as the oxygen adsorption and incorporation, as well as surface degradation are believed to be anisotropic. Therefore, precisely controlling the growth of single crystal thin film could be beneficial in understanding the complex function coupling between the ORR and the crystallographic orientation dependent surface states.

With the development of epitaxial film technology, thin film cathodes have been proposed as a tool to investigate intrinsic electrochemical activities [15]. Thin film cathodes with well-defined crystallographic orientation, low surface roughness, and a dense microstructure offer a comparable platform to further understand the ORR process [16,17]. Amongst all the possible thin film fabrication technologies, pulsed laser deposition (PLD) has been generally applied to prepare high quality complex oxide films due to its flexibility [18,19]. It has been previously used to prepare SOFC cathodes in both the fundamental studies and in practical applications [20].

In this paper, with the aim of shedding more light on the complex relationship between the ORR and the crystallographic orientation dependent surface states of SSNCF, we deposited using PLD, approximately 60 nm thick SSNCF oriented thin film electrodes on (l00) single crystalline 8 mol % Y_2_O_3_-stabilized ZrO_2_ (YSZ) substrates. YSZ substrates with a cubic structure and a = 5.14 Å were chosen due to their suitable lattice constant, good ionic conductivity, and negligible electronic conductivity, thereby allowing reliable transport measurements. The half in-plane lattice parameter was 3.63 Å along the [110] direction of the YSZ substrate, and considering the lattice parameter of SSNCF (cubic structure a = 3.92 Å), the in-plane mismatch was about 7%. The poor lattice match both in terms of crystal structure and lattice parameter could lead to a [110] direction growth, which has previously been reported for the deposition of La_0.8_Sr_0.2_CoO_3-δ_ and LaNiO_3_ on YSZ substrates [19,21]. To reorient the SSNCF growth direction; we grew the SSNCF thin film on the 10 nm thick 20 mol % Sm doped CeO_2_ (SDC, cubic structure a = 5.4 Å) buffered YSZ substrate. By introducing the SDC buffer layer, the in-plane mismatch between the SDC and SSNCF was reduced to about 3%. A better in-plane lattice parameter match could result in growth in the [001] direction.

## 2. Materials and Methods 

### 2.1. Powder and Target Preparation

SSNCF powders were prepared via a combined EDTA-citrate complexing method. Sr(NO_3_)_2_, Sc(NO_3_)_3_, Co(NO_3_)_2_·6H_2_O, Fe(NO_3_)_3_, and NbC_2_O_4_ were dissolved in water at the desired molar ratio. Next, the solution was mixed with EDTA-NH_3_ and citric acid to obtain a solution with a pH value adjusted to 7. After further evaporation at 80 °C for 5 h, the precursor was first calcined at 250 °C for 5 h, and then at 900 °C for 5 h in air to obtain SSNCF powder. The powders were collected and made into pellets under pressure of 14 MPa for the targets. Finally, round-shaped pellets of a 12 mm diameter size were sintered at 1200 °C for 5 h in air at a heating and cooling rate of 6 °C/min.

### 2.2. Thin Film Deposition

SSNCF films were grown by pulsed laser deposition (PLD) using the nanoPLD instrument from PVD Inc. (Wilmington, MA, USA). The KrF excimer pulsed laser source (COMPEX PRO 110 F, Cooherent LaserSystems GmbH & Co. KG., Göttingen, Germany) was operated at 10 Hz, with an energy density of 2 J/cm^2^; and the substrate temperatures were kept at 600 °C, 700 °C, and 800 °C during the thin film growth, respectively. The oxygen partial pressure was 50 mTorr, with a constant oxygen flux of 50 sccm. The distance from target to substrate was kept at 60 mm. For thin films of different orientations, two sets of the samples were prepared: (i) Fully covered films were prepared for the structural, morphological and electrical transport measuremnts. (ii) For the measurements of electrochemical performances, symmetrical SSNCF film electrodes were fabricated on the substrate. 

### 2.3. X-ray Diffraction 

The crystallographic quality and crystal structure of the SSNCF thin films were characterized by X-Ray diffraction (XRD) in the Bragg-Brentano geometry using a high resolution X-ray diffraction (HRXRD, Panalytical, Almelo, Netherlands) instrument. The X-ray source was Cu Kα (λ = 1.5406 Å). The θ–2θ measurements in the 2θ range of 20–60° were performed at 0.02° steps and an acquisition time of 0.2 s at each step, as well as characterizing the rocking curve in the range of ±1.5° near the corresponding characteristic peak. The working voltage and current of this instrument were set at 45 kV and 40 mA, respectively. 

### 2.4. Atomic Force Microscopy Characterization

The surface morphology of the thin films was characterized by atomic force microscopy (AFM). All topographic images were acquired under dry conditions in the tapping mode using silicon cantilevers at about 300 kHz through a Dimension Icon AFM with a Nanoscope V controller (Digital Instruments, Goleta, CA, USA).

### 2.5. Electrochemical Impedance Spectroscopy Measurement

The electrochemical impedance spectroscopy (EIS) measurements were accomplished by an electrochemical workstation (Bio-Logic, SP-300, Seyssinet-Pariset, France). Symmetrical SSNCF film electrodes with 2 mm × 5 mm dimensions were grown by PLD using a properly designed steel mask. The separation distance was 1 mm. The current was collected using Au wires bonded with Au paste on the two thin film electrodes. Measurements were performed in dry air with the flow rate of 100 sccm, and in the cooling ramp from 650 to 400 °C, with a temperature interval of 50 °C. Before the test, the temperature was first raised to 650 °C in 60 min, and then held at that level for one hour. All the samples were tested in the middle of the thermostatic section. Thereafter, a 100 mV AC voltage with a frequency range from 7 MHz to 10 MHz was applied.

### 2.6. DC Conductivity Measurement

The DC conductivity measurements (KEITHLEY, 2450 Source Meter, Cleveland, OH, USA) were performed via a Van-Der Pauw configuration. Four gold electrodes were painted on the edge of the sample with an average separation of 5 mm. The measurements were carried out in the temperature range of 400–650 °C during the heating ramp, and then repeated a few times to check the reliability of the results.

### 2.7. X-ray Photoelectron Spectroscopy 

The X-ray photoelectron spectroscopy (XPS) measurements were established using the Thermo Fisher ESCALAB 250XI (Fisher Scientific, Hampton, NH, USA). The ray source used was Al Kα radiation, 1486.74 eV energy which corresponded to an electron inelastic mean free path for the SSNCF samples of about 2.4 nm. For the single element analysis, the full spectrum of pass energy was 50 eV and 30 eV for the valence band spectra. The emission angle was 30°.

## 3. Results and Discussion

### 3.1. Thin Film Growth Optimization

**Figure 1:**
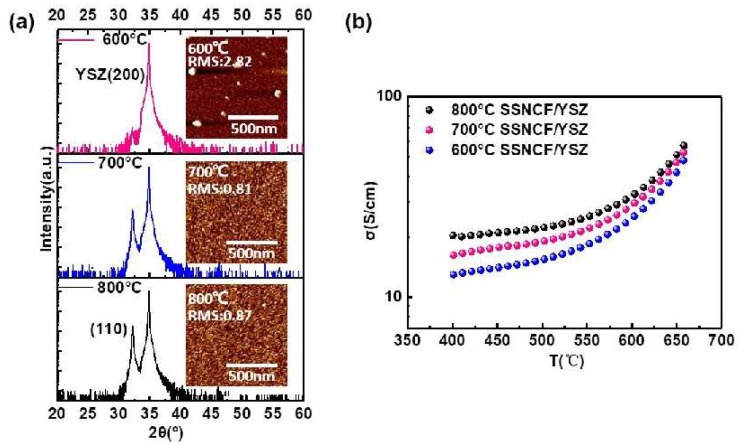
(**a**) X-ray diffraction spectra and atomic force microscopy (AFM) images of the SSNCF thin films grown at different temperatures. (**b**) Temperature-dependent electronic conductivities of the SSNCF thin films.

Using PLD, 60 nm thick SSNCF thin films were deposited on (*l*00)-oriented yttrium-stabilized zirconia (YSZ) substrates. The film thickness of the film grown at 800 °C was corrected by field emission scanning electron microscopy, and the thickness correction for the films grown at lower temperatures was performed using X-ray reflectivity measurements. (In Supplementary Materials Section S1 for field emission scanning electron microscopy images and for the X-ray reflectivity measurements). The growth temperature of the SSNCF thin films was optimized at 600 °C, 700 °C, and 800 °C. The XRD patterns are shown in Figure 1a. The sample prepared at 600 °C showed no characteristic peak of the SSNCF thin film, and indicated a poor crystallinity of the samples. For the films grown at 700 °C and 800 °C, both films showed a diffraction peak near 32°, which could be assigned to the (110) reflection of the SSNCF. The lattice parameter of the thin films was calculated to be 3.93 Å, similar to its bulk value [11,12]. We further compare the surface quality of the SSNCF thin films prepared at different temperatures by atomic force microscopy (AFM). From the AFM images shown in the inset of Figure 1a, the film deposited at 600 °C shows agglomerated large grains with the highest surface roughness. This may have been due to the slow surface nucleation process and growth kinetics at lower temperatures [10]. The surface roughness decreased from 2.82 nm at a growth temperature of 600 °C to 0.87 nm at a temperature of 800 °C. 

The results of the temperature dependent electronic conductivity measurements are shown in Figure 1b. The measurements were performed at a temperature range of 650 °C to 400 °C in air. The overall electronic conductivity increased as a function of the temperature, indicating a thermal activated semiconducting hopping behavior [22]. The conductivities improved with the increasing thin film deposition temperature over the whole temperature range. For example, at 650 °C, the electronic conductivity increased from 48 S/cm for the SSNCF film grown at 600 °C to 57 S/cm deposited at 800 °C. Based on the results of the crystal structural, morphological, and electronic transport characterization, the thin film grown at 800 °C exhibited good crystallographic quality, low surface roughness, and acceptable electronic conductivity. Therefore, we choose 800 °C as the deposition temperature to fabricate SSNCF thin film electrodes for further electrochemical characterization.

### 3.2. Structure Reorientation with a Buffer Layer

**Figure 2:**
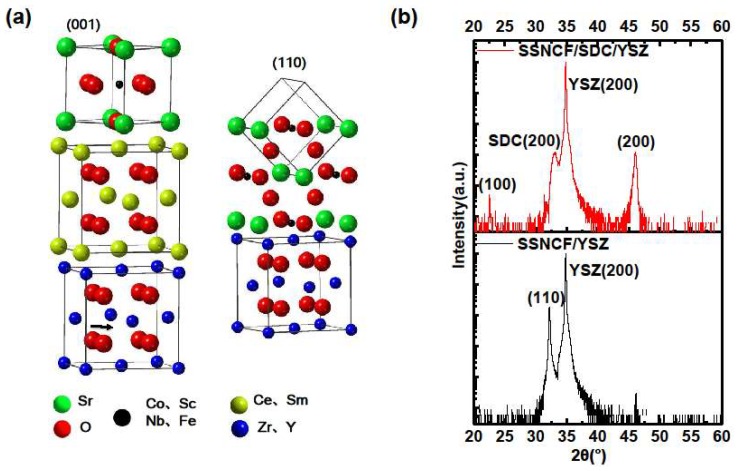
(**a**) Schematic sketch of the correlation between the SSNCF, SDC, and YSZ of different oriented SSNCF thin films. (**b**) X-ray diffraction spectra of the SSNCF thin films on the substrate with or without the SDC buffer layer.

To influence the growth orientation of SSNCF, a 10 nm thick SDC buffer layer was introduced onto the (*l*00)-oriented YSZ substrate before fabricating the SSNCF thin film. The better in plane lattice parameter match led to the growth of the SSNCF thin film in the [100] direction. The SDC is an oxygen-ion conductor with similar thermal expansion coefficient and ionic conductivity to the YSZ [23,24]. The ultra-thin SDC buffer layer can reorient the SSNCF growth direction without significantly modifying the oxygen ion conductivity of the YSZ single crystal substrate. The epitaxial relationships are demonstrated in the Figure 2a. Typical X-Ray scans of the SSNCF/YSZ sample and the SSNCF/SDC/YSZ sample are shown in Figure 2b. Only the (*l*00) reflections from SSNCF were visible in the SSNCF/SDC/YSZ, suggesting that the film was highly textured along the [100] direction. In the case of the SSNCF/YSZ, the presence of only the (110) reflection demonstrated the SSNCF thin film growing along the [110] direction. Such observation proofs that the SDC buffer layer could be used to adjust the film growth, allowing us to directly investigate the growth orientation dependent SSNCF’s ORR activity. To rule out the possible overlap presented by the SDC buffer layer and SSNCF, we carefully compared the XRD pattern of the SDC/YSZ and SSNCF/SDC/YSZ, as seen in Supplementary Materials Section S4. Only the (l00) peaks of the SDC were observed, indicating a good crystallinity of SDC buffer layer. More importantly, the SDC diffraction peaks were almost identical without any peak width broadening and intensity decrement after the deposition of the SSNCF layer, which was extremely help for us in ruling out the presence of (110)-oriented grains of the SSNCF layer. We calculated the lattice parameters of both samples using Bragg’s equation. The lattice parameter of the SSNCF/YSZ thin film was 3.93 Å, while the lattice parameter of the SSNCF/SDC/YSZ was 3.95 Å. The films crystallographic quality was examined by Rocking curve measurements. The SSNCF grown directly on the YSZ demonstrated a smaller half width at half maximum than on the SDC buffered YSZ, as shown in Supplementary Materials Section S2. Such results indicated that a better crystallographic quality was achieved for the SSNCF/YSZ. 

### 3.3. ORR Activity

**Figure 3:**
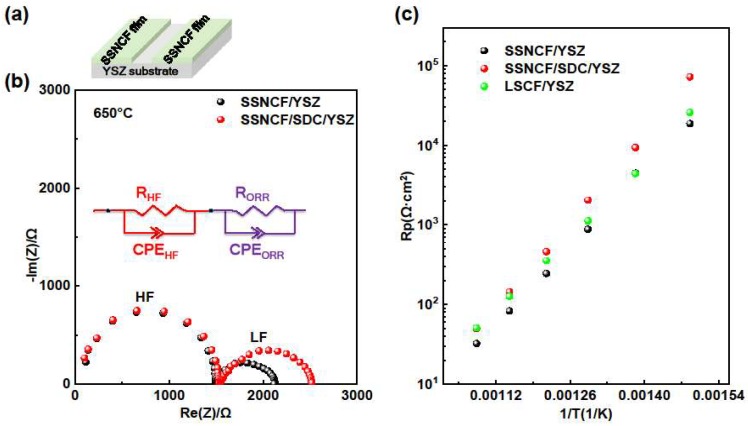
(**a**) Sketch of sample the SSNCF thin film on the YSZ substrate (**b**) Nyquist plots of the SSNCF thin films measured at 650 °C in air, with the equivalent circuit inset. (**c**) The Arrhenius type Rp plots of the SSNCF thin films and reference LSCF thin film.

To study the oxygen reduction reaction (ORR) performance of the cathode, the electrochemical impedance spectra (EIS) measurements were performed at a range of 400 °C to 650 °C in air. The sample setup is shown in Figure 3a. Two strips of SSNCF thin film electrodes were deposited on YSZ and SDC buffered YSZ substrates, respectively. The Nyquist plots of the SSNCF/YSZ and SSNCF/SDC/YSZ thin film electrodes obtained from the EIS measurement at 650 °C are shown in Figure 3b. Both Nyquist plots showed two semicircles. The impedance spectrum was further divided into a high frequency (HF) semicircle and a low frequency (LF) one, as indicated in Figure 3b. The HF semicircle was related to the limited oxygen ion conductivity of the YSZ and SDC buffered YSZ substrates. As expected, the 10 nm thick SDC buffer layer did not introduce any observable variation in the Nyquist plot. On the other hand, the LF semicircle was linked to the ORR polarization process.

The plots were fitted by two RC equivalent circuits in series, which is the inset of Figure 3b. The fitting details could be found in Supplementary Materials Section S3. The polarization resistance (Rp) estimated from the LF part varied with the thin films’ crystallographic orientation. At 650 °C, the Rp value of the SSNCF/YSZ and SSNCF/SDC/YSZ were 30.9 Ω·cm^2^ and 45.6 Ω·cm^2^, respectively. The SSNCF thin film electrodes grown along the [110] had lower R_p_, suggesting a superior ORR activity compared to the sample grown along the [100] direction.

Figure 3c gives the Arrhenius type R_p_ plots of the SSNCF/YSZ and SSNCF/SDC/YSZ thin film electrodes. The R_p_ of the SSNCF/YSZ was smaller than the SSNCF/SDC/YSZ in the whole temperature range, which could either be due to a better crystalline quality and/or different surface chemical states. The activation energy (E_a_) obtained from the R_p_ plots was 1.45 eV for the SSNCF/YSZ and 1.62 eV for the SSNCF/SDC/YSZ sample. The fitting equation was $R_{p}=R_{p}^{0}\exp\left(-\frac{\mathrm{Ea}}{K_{B}T}\right)$ as described by H. Tuller et al. [25]. Such values are typical for MIEC electrodes, as previously reported for La_1−x_Sr_x_CoO_3−__δ_ and Ba_1−x_Sr_x_Co_1−y_Fe_y_O_3−__δ_ [4]. The ORR activity of the SSNCF thin film electrodes were compared with the state-of-the-art La_0.6_Sr_0.4_Co_0.8_Fe_0.2_O_3−__δ_ (LSCF) thin film electrodes with the same thickness on the (100) oriented YSZ substrate. Both SSNCF thin film electrodes showed similar Rp compared to the LSCF, suggesting that the SSNCF thin film electrodes may be considered as a potential cathode material for IT-SOFCs.

**Figure 4:**
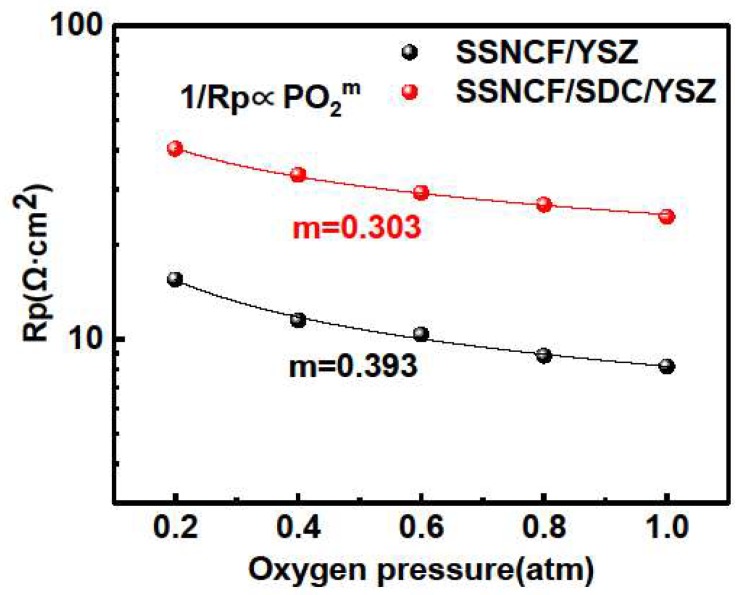
The pO2 dependent Rp of the SSNCF thin film electrodes grown on the YSZ- and SDC-buffered YSZ substrates at 650 °C with partial pressure of oxygen in the range 1–0.1 atm.

The rate-determining step of the ORR process was investigated by the pO_2_ dependent EIS measurements at 650 °C. The dependence of the Rp on the partial pressure of oxygen generally demonstrated the following $R_{P}\propto P_{O_{2}}^{-m}$ behavior. The exponential factor *m* can be used to describe the kinetic rate-determining step during the ORR process [25]. When *m* equals to 0.25, the reaction kinetics are controlled by oxygen dissociation, and if *m* equals to 0.5, the charge transfer process occurring at the air-electrode or electrode-electrode interface is the most crucial step for the reaction kinetics [10]. The pO_2_-dependent EIS results can be found in Figure 4. Both the SSNCF/YSZ and SSNCF/SDC/YSZ samples demonstrate larger Rp values at a lower partial pressure of oxygen, confirming their cathodic nature. In addition, the *m* values obtained from fitting the experimental data with the above equation were 0.393 for the SSNCF/YSZ and 0.303 for the SSNCF/SDC/YSZ. Such values suggested that both of them may have included both the oxygen adsorption/dissociation and the charge transport contributions. The increment of m could indicate the rate-determining step shifts from oxygen adsorption/dissociation for the SSNCF/YSZ thin film and to charge transport for the SSNCF/SDC/YSZ. 

### 3.4. Surface Sensitive X-Ray Photoelectron Spectroscopy

To further understand the significant crystallographic orientation dependent ORR activity and to make connections to their surface states. Surface-sensitive X-Ray photoelectron spectroscopy (XPS) was applied to the SSNCF/YSZ and SSNCF/SDC/YSZ thin films. The Co valence state and surface chemistry were determined by measuring the Co, O, and Sr core levels. The dopants showed relatively poor signal to noise ratio due to their limited spatial resolution. Therefore, the core level spectra of the dopants were not conducted with the multicomponent fitting analysis.

**Figure 5:**
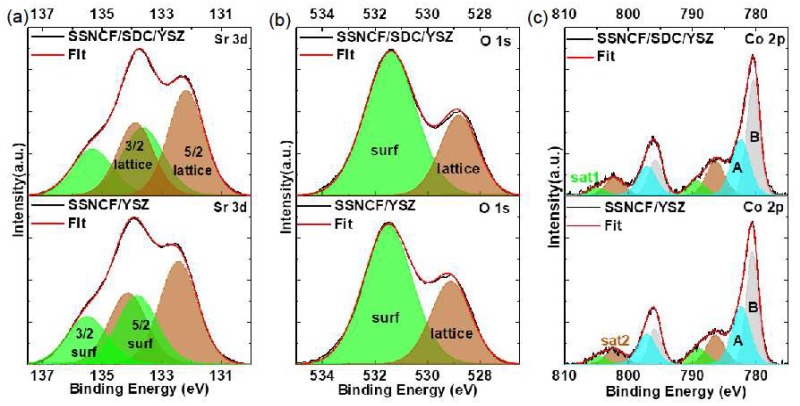
X-ray photoelectron spectroscopy (XPS) and fitted data of Sr, O and Co core level spectra.

Figure 5 shows the XPS results and fitting information for the three elements Sr, O, and Co The report from Crumlin et al. has linked ORR activity to the surface Sr content [26]. The surface Sr enrichment, typically in the form of Sr based oxide, carbonates, and hydroxide, has been considered as one of the main reasons for surface deactivation of the ORR. As observed in the Figure 5a,b the Sr 3d and O 1s spectra had different shapes for the SSNCF/YSZ and SSNCF/SDC/YSZ samples. Based on our previous work on La_1−x_Sr_x_CoO_3−__δ_ thin films, the Sr 3d can be deconvoluted into “surface” and “lattice” components [15]. Each component contains a doublet of spin orbit components with a splitting energy of 1.4 eV [27]. The low energy doublet (${\mathrm{BE}}_{5/2}=132.4\mathrm{eV}$) is attributed to the lattice component in the SSNCF, named as “lattice”. The high energy doublet (${\mathrm{BE}}_{5/2}=133.8\mathrm{eV}$) was assigned to the Sr surface species, named as “surface”. The O 1s spectrum could be deconvoluted into the corresponding “surface” and “lattice” components as well. The low energy “lattice” component at 529.1 eV was related to the oxygen in the SSNCF lattice, and the high energy “surface” at 531.4 eV could be assigned to the Sr enriched surface species [28]. 

A noticeable decrease of “surface” and “lattice” ratio of Sr 3d spectra to 0.74 for the SSNCF/SDC/YSZ and 0.71 for the SSNCF/YSZ thin film could be obtained. A similar trend was observed in O 1s spectra, that is the “surface” and “lattice” ratio decreased from 2.44 for the SSNCF/SDC/YSZ to 2.08 for the SSNCF/YSZ sample. Such results were in agreement with the orientation dependent ORR activity of the SSNCF thin film electrodes. The stronger Sr enrichment for the SSNCF/SDC/YSZ may lead to a reduction in the ORR activity. The stronger Sr enrichment could be due to the worsening crystallographic quality of the SSNCF/SDC/YSZ compared to the SSNCF/YSZ, as shown in Supplementary Materials Section S2. The possible crystal defects could lead to a more facile Sr transport during growth and thus, a greater Sr enrichment at the surface.

The spectra of Co 2p for the SSNCF/SDC/YSZ and SSNCF/YSZ thin films are shown in Figure 5c, from which the 2p_1/2_ and 2p_3/2_ spin-orbit doublet can be found. Owing to the presence of the screening effect, the exact determination of the cobalt valence state from the main core level peaks was quite challenging. However, it is well accepted the Co 2p_3/2_ binding energy can be used to determine the cobalt ion valence state: the binding energy increases from cobalt (II) to cobalt (III). Such a shift is due to the increase in the effective charge of the cobalt metallic center [29]. In Figure 5c it was found that the Co*2p*_3/2_ binding energy increased from 780.5 eV for the SSNCF/SDC/YSZ to 780.6 eV for the SSNCF/YSZ. This observation indicated that the SSNCF thin film grown along the [110] direction could possess a higher cobalt ion oxidation state, which is believed to be essential for enhancing the ORR activity [30,31]. It should be noted that the spectra contained “shake-up” satellites (Sat 1 and Sat 2), as shown in Figure 5c. Sat 1, which was 8 eV higher than the main component at 797 eV, could be assigned to the charge transfer from the O 2p to Co^3+^ and Co^4+^ 3d orbitals. The Sat 2 with a peak position 6 eV higher than the main component was generally attributed to cobalt (II) with high-spin state. Therefore, the cobalt ion oxidation state variation could also be evaluated from the ratio of Sat 1 to Sat 2. The larger the ratio is, the higher the cobalt ion oxidation state. This observation was in agreement with [31] that a stronger surface segregation could lead to a reduction in the oxidation state of cobalt ion due to a reduced oxygen content. The ratio increased from 0.43 for SSNCF/SDC/YSZ to 0.55 for SSNCF/YSZ, supporting the observation obtained from the Co 2p_3/2_ main component. That is, the (110) oriented SSNCF thin film electrodes cobalt ion oxidation state is higher than (100) oriented SSNCF thin film electrodes. From these surface sensitive XPS measurements, lower Sr enriched surface species and higher cobalt ion oxidation states observed for the SSNCF/YSZ were helpful in explaining the improved ORR activities. 

## 4. Conclusions

In this work, 60 nm thick SSNCF thin films with good crystallinity and flat surface roughness were prepared using PLD at 800 °C. The thin film on the YSZ substrate and on the SDC/YSZ substrate had different growing directions, being the [110] and [100], respectively. From the current investigation, the SDC buffer layer proved to be valid for adjusting the growth direction of the SSNCF thin film. The ORR activities of the SSNCF thin film electrodes with different crystallographic orientations were compared using EIS measurements in air. The film grown along the [110] direction demonstrated lower polarization resistances and smaller activation energy than the film grown along [100] direction, indicating a better ORR activity. A comparison with the state-of-the-art LSCF thin film electrode suggested that the SSNCF film could be a good potential candidate as a cathode working in the intermediate temperature range. The crystallographic orientation dependence of the ORR activities was linked to surface chemistry through XPS characterization. It was found that lower Sr-enrichment and higher cobalt-ion oxidation states were beneficial for the ORR process. This investigation of the SSNCF thin films with different crystallographic orientation can be helpful in taking a step forward in the optimization of thin films’ electrochemical activity through tunable crystallographic orientation.

## Supplementary Materials

The following are available online at https://www.mdpi.com/1996-1944/12/5/777/s1, Figure S1: FE-SEM micrograph (cross-section) of LSCO/YSZ film, Figure S2: XRR patterns of thin films prepared at 700 and 600 °C, Table S1: Roughness and projected difference on the surface area for the SSNCF films grown on the YSZ and SDC/YSZ substrates with different temperatures, Figure S3: Atomic force microscopy of SSNCF/YSZ and SSNCF/SDC/YSZ, Figure S4: Rocking curves of SSNCF thin films with different orientation, Figure S5: (a) Nyquist plots of the LSCF and SSNCF samples in air at 650 °C with the equivalent circuit used to fit data. (b) The temperature dependent polarization resistance (Rp) in a logarithmic scale of different thin films in dry air from 400 °C to 650 °C, Figure S6: Nyquist plots of the before and after regulation SSNCF film electrodes under different temperatures, Table S2: Fitting results of the SSNCF/YSZ electrode, Figure S7: Nyquist plots of the SSNCF and LSCF film electrodes under different temperatures, Table S3: Fitting results of the SSNCF/SDC/YSZ electrode. Figure S8: XRD pattern comparison of SDC/YSZ and SSNCF/SDC/YSZ.
