# Supplementary material for: Textured Sr2Sc0.1Nb0.1Co1.5Fe0.3O6−2δ Thin Film Cathodes for IT-SOFCs
Source: Materials (Basel). 2019 Mar 7;12(5):777. doi: 10.3390/ma12050777 (PMC6427658; doi:10.3390/ma12050777)
Supplement: Supplementary file 1 [file materials-12-00777-s001.pdf]

materials

*Supplementary*

# Textured $Sr_2Sc_{0.1}Nb_{0.1}Co_{1.5}Fe_{0.3}O_{6-2\delta}$ Thin Film Cathodes for IT-SOFCs

**Zhaoxin Zhu [1,2,3], Chuan Zhou [4], Wei Zhou [4,*] and Nan Yang [1,*]**

[1] School of Physical Science and Technology, ShanghaiTech University, 393 Middle Huaxia Road, Pudong, Shanghai 201210, China
[2] Shanghai Institute of Ceramics, Chinese Academy of Sciences, Shanghai 200050, China
[3] University of Chinese Academy of Sciences, Beijing 100049, China
[4] Jiangsu National Synergetic Innovation Center for Advanced Materials (SICAM), State Key Laboratory of Materials-Oriented Chemical Engineering, College of Chemical Engineering, Nanjing Tech University, No.5 Xin Mofan Road, Nanjing 210009, China
* Correspondence: yangnan@shanghaitech.edu.cn (N.Y.); zhouwei1982@njtech.edu.cn (W.Z.)

Received: 15 January 2019; Accepted: 24 February 2019; Published: 7 March 2019

## S1. SEM details (scanning electron microscope)

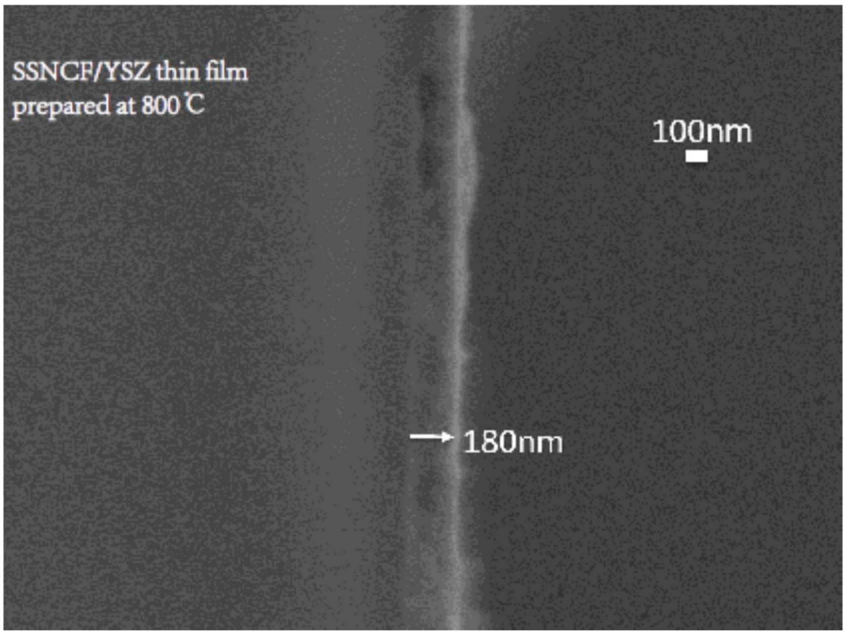


**Figure S1.** FE-SEM micrograph (cross-section) of LSCO/YSZ film.

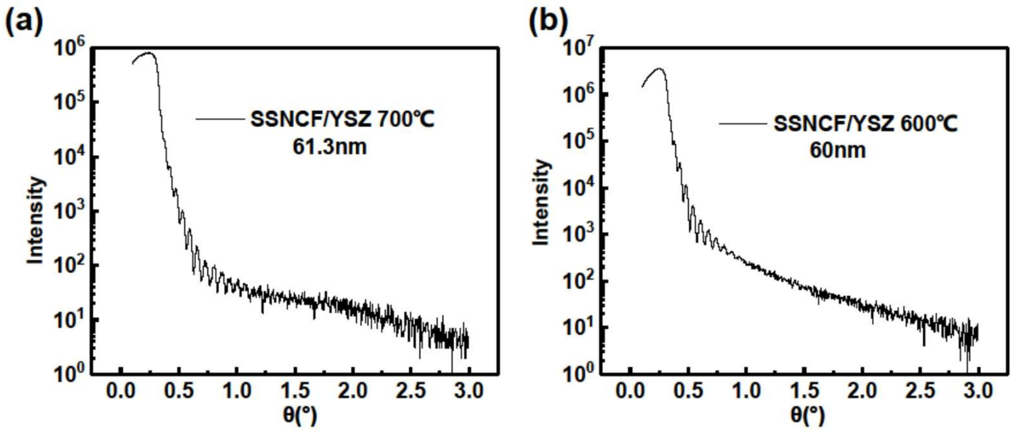

**Figure S2.** XRR patterns of thin films prepared at 700 and 600 °C.

Field emission scanning electron microscopy (FE-SEM) microscopy was performed using a JSM7800 microscope with an LED (low level electron detector) with 6000 times magnification and 5 kV acceleration voltage. Figure S1 shows an FE-SEM micrograph of the surface crack of the SSNCF thin film sample prepared at 800 °C. The thickness of this sample is about 180 nm, the number of depositions is 9000, and the other samples in this experiment are all 3000 shots, which is roughly consistent with our deposition rate estimation. The cross-sectional microstructure can verify the uniformity of the film sample. Further, thickness of the thin films prepared at other temperatures can be verified using the X-ray reflection (XRR) in Figure S2. All the thicknesses of the thin films are around 60 nm.

## S2. Morphological characterization and rocking curve

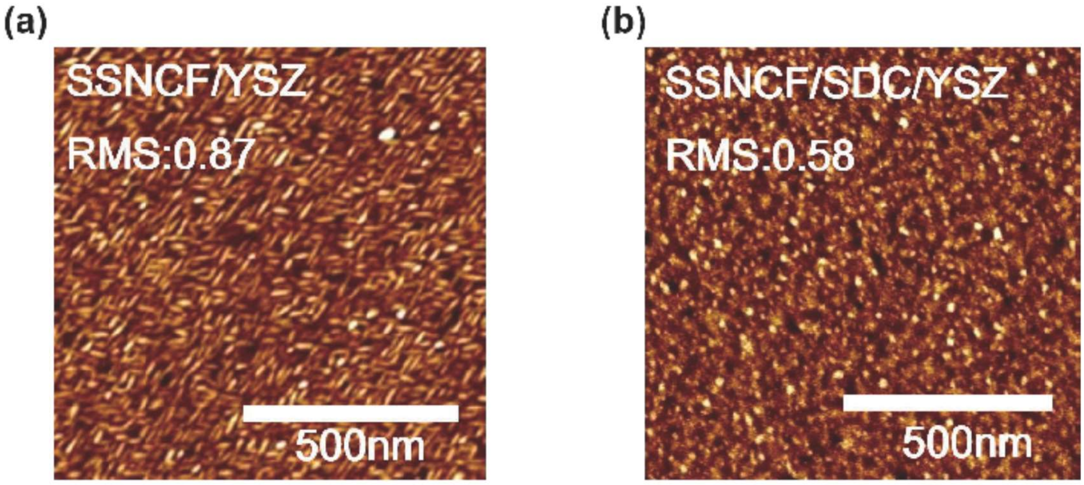


**Figure S3.** Atomic force microscopy of SSNCF/YSZ and SSNCF/SDC/YSZ.

A typical form of the SSNCF film is shown in Figure S3. The scan size is 1 μm × 1 μm. The film exhibited a dense and uniform surface morphology. The values of Rq (root mean square roughness) and Ra (arithmetic mean roughness) are shown in Table S1. It can be observed that the SSNCF film has a flatter surface topography at 800°C. The surface of the SSNCF grown directly on the YSZ substrate and the SDC/YSZ substrate is very flat, and the surface roughness of the SSNCF/SDC/YSZ is even lower.

**Table S1.** Roughness and projected difference on surface area for SSNCF films grown on YSZ and SDC/YSZ substrates with different temperatures.

| Growth conditions | Rq (nm) | Ra (nm) |
|---|---|---|
| SSNCF/YSZ_800 °C | 0.868 | 0.686 |
| SSNCF/YSZ_700 °C | 0.805 | 0.638 |
| SSNCF/YSZ_600 °C | 2.82 | 1.73 |
| SSNCF/SDC/YSZ_800 °C | 0.582 | 0.449 |

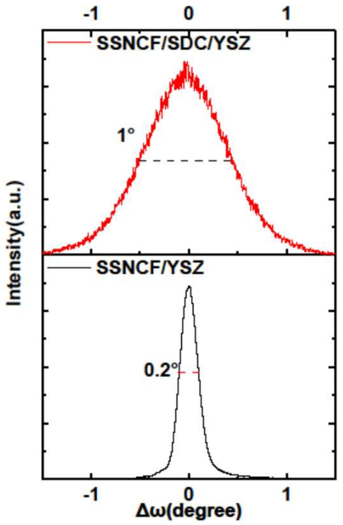


**Figure S4.** Rocking curves of SSNCF thin films with different orientation.

The rocking curve scans for SSNCF/YSZ and SSNCF/SDC/YSZ films are shown in Figure S4. The half width at half maximum (FWHM)-peak width are 0.2° and 1° for SSNCF/YSZ and SSNCF/SDC/YSZ films, respectively. Because the FWHM of rocking curve is inverse proportional to the crystallinity of thin film. A better crystallographic quality is achieved for SSNCF/YSZ.

## S3. Electrochemical Impedance spectroscopy (EIS) and Electronic conductivity

The Nyquist plots of SSNCF/YSZ and LSCF/YSZ thin film electrodes obtained from EIS test at 650 °C in air are shown in Figure S5 (a). In Figure S5 (b), it can be seen that the SSNCF / YSZ thin film electrodes has a smaller polarization resistance (Rp) value and a close activation energy than the LSCF / YSZ thin film electrodes.

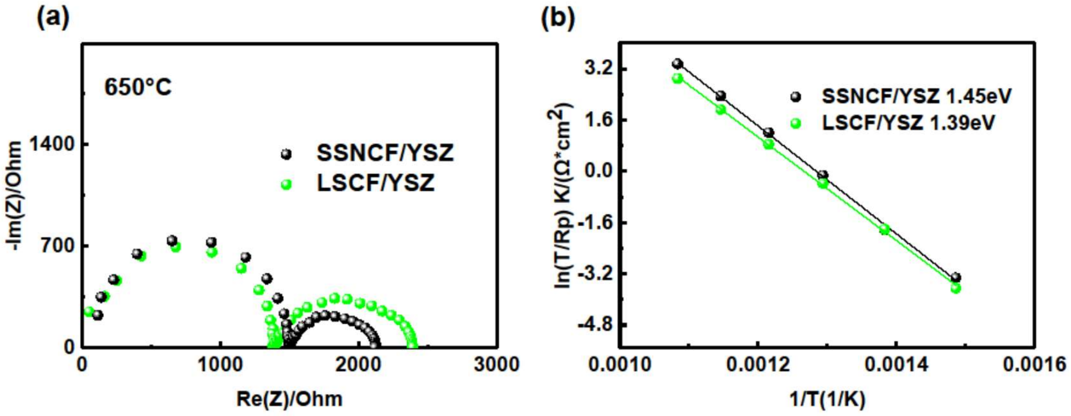


**Figure S5.** (**a**) Nyquist plots of LSCF and SSNCF samples in air at 650 °C with the equivalent circuit used to fit data. (**b**) The temperature dependent polarization resistance (Rp) in logarithmic scale of different thin films in dry air from 400 °C to 650 °C.

The effect of the different orientations of the thin film electrodes on the polarization resistance was evaluated by EIS measurement as a function of temperature in order to obtain the activation energy from the Arrhenius pattern. Two SSNCF electrodes with a single distance of 1 mm were grown by PLD using a steel mask. Thus, the sample geometry can be described as a SSNCF $_{\text{film electrode}}$ / YSZ $_{\text{substrate electrolyte}}$/ SSNCF $_{\text{film electrode}}$ film electrode. Then we used Au paste as a current collector and painted on the SSNCF thin film electrodes. The samples geometry can be observed in Figure 3(a) of the manuscript. Measurements were taken at 650 °C to 400 °C using a Bio-Logic SP-300

electrochemical workstation. The temperature step is 50°C, the measured drive amplitude is 100 mV, and the drive frequency is 7 MHz to 10 mHz [1].

Figure S6 shows typical Nyquist plots of the SSNCF film electrodes at each test temperatures. The fitting circuit is shown in the insertion part of Figure 3(b).

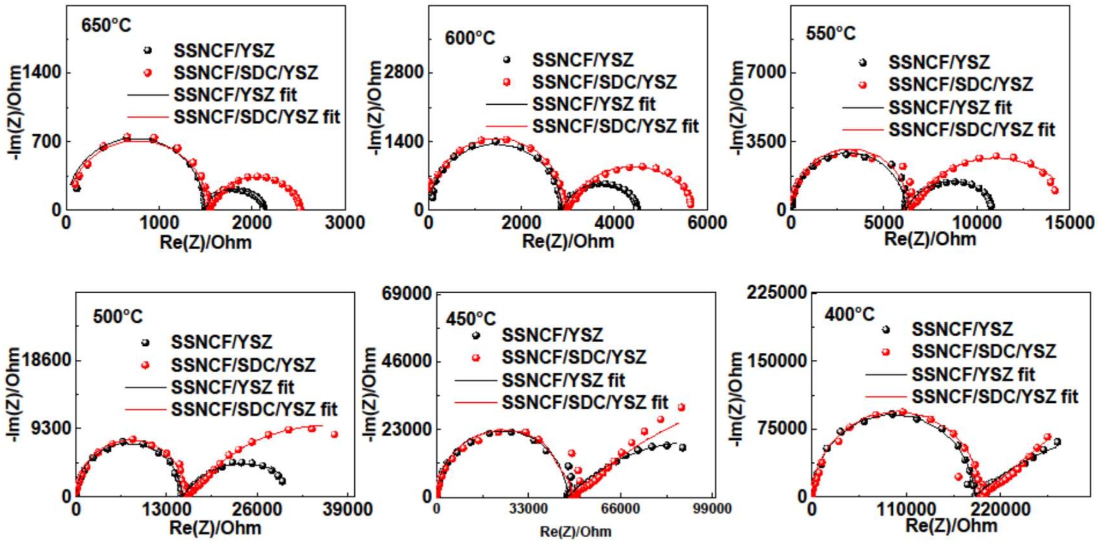


**Figure S6.** Nyquist plots of before and after regulation SSNCF film electrodes under different temperatures.

These data are then fitted with an equivalent circuit consisting of two parallel RQ circuits in series as described in the following: (parallel R1, Q1) and (parallel R2, Q2), where the 1 refers to the high frequency element (R1 and Q1) which is related to the limited oxygen ion conductivity of YSZ and SDC buffered YSZ substrates. 2 refers to the low frequency component due to the electrode polarization, which is linked to the ORR polarization process. The fitting information is shown in Table 2 and Table 3 [2].

**Table S2.** Fitting results of SSNCF/YSZ electrode.

| T(K) | Q1 | a1 | R1(Ω) | Q2 | a2 | R2(Ω) |
|---|---|---|---|---|---|---|
| 923 | 8.35E-11 | 1 | 1489 | 1.90E-04 | 0.7678 | 646.3 |
| 873 | 1.38E-10 | 0.9653 | 2878 | 1.70E-04 | 0.7418 | 1660 |
| 823 | 1.22E-10 | 0.9736 | 6181 | 1.42E-04 | 0.6944 | 4906 |
| 773 | 1.14E-10 | 0.9767 | 15260 | 9.78E-05 | 0.6088 | 17573 |
| 723 | 1.04E-10 | 0.9856 | 46901 | 5.57E-05 | 0.5022 | 89343 |
| 673 | 1.10E-10 | 0.9792 | 189250 | 2.47E-05 | 0.4594 | 374175 |

The comparisons of SSNCF thin films and LSCF thin films of Nyquist plots are shown in Figure S7. The polarization resistance ($R_P$) indicated from LF part varies with the selection of different thin film materials. At 650 °C, the $R_P$ value of SSNCF/YSZ and LSCF/YSZ are 30.9 $\Omega\cdot cm^2$ and 50.1 $\Omega\cdot cm^2$ respectively. The SSNCF/YSZ thin film electrodes with the growth direction along [110] has lower $R_P$, leading to better ORR activity compared with the LSCF/YSZ thin film grown along [110] direction.

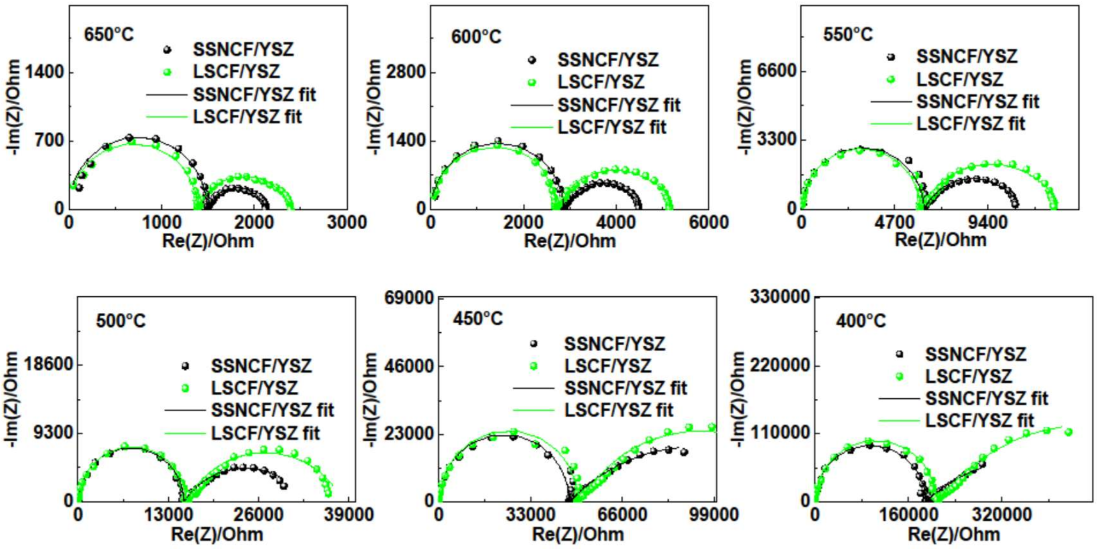


**Figure S7.** Nyquist plots of SSNCF and LSCF film electrodes under different temperatures.

**Table S3.** Fitting results of SSNCF/SDC/YSZ electrode.

| T(K) | Q1 | a1 | R1(Ω) | Q2 | a2 | R2(Ω) |
|---|---|---|---|---|---|---|
| 923 | 1.68E-10 | 0.9555 | 1543 | 2.36E-04 | 0.765 | 997.9 |
| 873 | 6.27E-11 | 1 | 2949 | 2.23E-04 | 0.7073 | 2887 |
| 823 | 7.24E-11 | 0.9938 | 6362 | 1.84E-04 | 0.6702 | 9210 |
| 773 | 7.23E-11 | 0.9942 | 15808 | 1.27E-04 | 0.567 | 40890 |
| 723 | 1.08E-10 | 0.9694 | 48381 | 6.60E-05 | 0.4781 | 186878 |
| 673 | 1.03E-10 | 0.9743 | 197557 | 3.32E-05 | 0.4681 | 1.44E+06 |

## S4. XRD pattern comparison of SDC/YSZ and SSNCF/SDC/YSZ

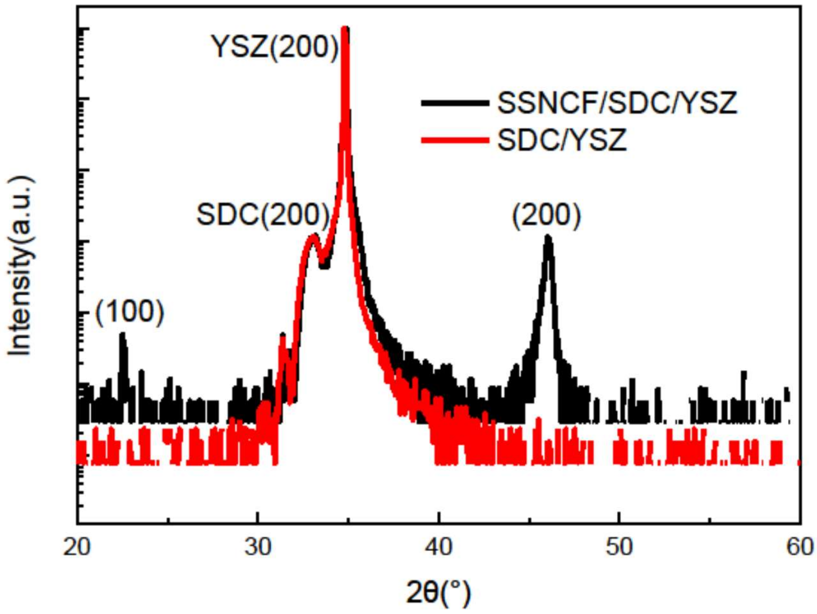


**Figure S8.** XRD pattern comparison of SDC/YSZ and SSNCF/SDC/YSZ.

The XRD of SDC grown on YSZ is shown in the red line part in Figure S8, and the deposited SSNCF/SDC/YSZ is shown in the black line part of the Figure S8. The SDC diffraction peaks are

almost the same, without any peak width broadening and strength reduction after deposition of the SSNCF layer.

## References

1. Zhu, Z.; Shi, Y.; Aruta, C.; Yang, N. Improving Electronic Conductivity and Oxygen Reduction Activity in Sr-Doped Lanthanum Cobaltite Thin Films: Cobalt Valence State and Electronic Band Structure Effects. *ACS Appl. Energy Mater.* **2018**, *1*, 5308–5317, 10.1021/acsaem.8b00931, doi:10.1021/acsaem.8b00931.
2. Baumann, F.S.; Maier, J.; Fleig, J. The polarization resistance of mixed conducting SOFC cathodes: A comparative study using thin film model electrodes. *Solid State Ionics* **2008**, *179*, 1198–1204, doi:10.1016/j.ssi.2008.02.059.

© 2019 by the authors. Submitted for possible open access publication under the terms and conditions of the Creative Commons Attribution (CC BY) license (http://creativecommons.org/licenses/by/4.0/).
